# 

**Published:** 1909-03

**Authors:** 


### LITERARY NOTES

SAUNDERS'S Books is the title of a new descriptive catalogue of medical and surgical works issued by the W. B. Saunders Company, medical publishers, Philadelphia and London. It is handsomely printed in tinted paper, fully illustrated, and is a beautiful specimen of the printing and engraving arts. It contains a description of almost every book a physician really reads and may be obtained on application to the publishers, 925 Walnut Street, Philadelphia.

---

THE *American Journal of Surgery,* New York, announces that Dr. James P. Warbasse, late editor N. Y. State Medical Journal, has joined its editorial staff. The March number will contain articles by Lilienthal, Young, Simpson, Hopkins, Tuttle, Brickner, Warbasse, Bandler, Meyer, Hartwell, deLatour, Sellings, and others.

